# A Preliminary Cost-Utility Analysis of the Prosthetic Care Innovations: Basic Framework

**DOI:** 10.33137/cpoj.v4i2.36365

**Published:** 2021-09-21

**Authors:** L Frossard

**Affiliations:** 1 YourResearchProject Pty Ltd, Brisbane, Australia.; 2 Griffith University, Gold Coast, Australia.; 3 University of the Sunshine Coast, Maroochydore, Australia.; 4 Queensland University of Technology, Brisbane, Australia.

**Keywords:** Artificial Limbs, Bionic Limbs, Bone-Anchored Prosthesis, Cost-Effectiveness, Cost-Utility, Health Economic Evaluation, Health Technology Assessment, Prosthesis, Socket-Suspended Prosthesis

## Abstract

A preliminary cost-utility analysis (CUA) of prosthetic care innovations can provide timely information during the early stage of product development and clinical usage. Concepts of preliminary CUAs are emerging. However, several obstacles must be overcome before these analyses are performed routinely. Disparities of methods and high uncertainty make the outcomes of usual preliminary CUAs challenging to interpret, appraise and share. These shortcomings create opportunities for a basic framework of preliminary CUAs. First, I introduced a basic framework of a preliminary CUA built around a series of constructs and hands-on recommendations. Then, I appraised this framework considering the strengths and weaknesses, barriers and facilitators, and return on investment. The design of the basic framework was determined through the review of health economic and prosthetic-specific literature. A preliminary CUA comparing the costs and utilities between usual intervention and an innovation could be achieved through a 15-step iterative process focusing on feasibility, constructs, analysis, and interpretation of outcomes. This CUA provides sufficient evidence to identify knowledge gaps and improvement areas, educate about the design of subsequent full CUAs, and obtain fast-track approval from governing bodies. Like previous CUAs, the main limitations were inherent to the constructs (e.g., narrow perspective, plausible scenarios, mid-term time horizon, substantial assumptions, data mismatch, high uncertainty). Key facilitators potentially transferable across preliminary CUAs of prosthetic care innovations included choosing abided constructs, capitalizing on prior schedules of expenses, and benchmarking baseline or incremental utilities. This new approach with preliminary CUA can simplify the selection of methods, standardize outcomes, ease comparisons between innovations, and streamline pathways for adoption. Further collegial efforts toward validating standard preliminary CUAs will facilitate access to economic prosthetic care innovations, improving the lives of individuals suffering from limb loss worldwide.

## INTRODUCTION

The revolutionary car maker and industrialist Henry Ford (1863-1947) said, “*if you think of standardization as the best that you know today, but which is to be improved tomorrow; you get somewhere*.” The automobile and healthcare industries might be two worlds apart. However, they both thrive on standardization because it is the key to efficiency and safety. Hence, efforts are needed to develop a standard of care. Standardization could also be applied to assess the socio-economic benefits of prosthetic care intervention. This article focuses on developing a basic framework of preliminary cost-utility analysis (CUA) of innovations suitable to improve prosthetic fittings.

### Economic evaluations of prosthetic care innovations

Promoters of prosthetic care interventions, including end-users, providers of prosthetic solutions, and administrators of healthcare organizations, are increasingly motivated to demonstrate the socio-economic benefits of their innovations.^1–3^

Either health technology assessment (HTA) or health economic evaluation (HEE), or both of prosthetic care innovations are imperative to systematically assess the indirect and unintended clinical and economic consequences of an intervention.^4–6^ Practically, there is an ever-increasing demand for CUAs comparing the usual and new interventions using the incremental cost-utility ratio (ICUR). The ICUR is based on the incremental costs expressed in monetary units and utilities expressed in quality-adjusted life-year (QALY) over time compared to the willingness-to-pay threshold (WTP).^4–9^

As detailed in Frossard (2021), early, preliminary, and full CUAs can be conducted at the early, middle, and late phases of product development and clinical acceptance of innovations, respectively.^3^

Full CUAs, including primary and modeling analyses, can produce comprehensive outcomes, but they require substantial resources and lack timeliness. Promoters could rely on strong recommendations that might be provided after a wide clinical adoption. Full CUAs have been used to demonstrate the health economic benefits of socket fitting interventions and fitting of advanced prosthetic components (microprocessor-controlled knees, energy-storing, and return feet) for socket-suspended and socket-free prostheses.^7,8,10–19^

Earlier CUAs could be conducted around the initial and middle stages of innovation development when clinical usage is still limited. These analyses could provide timely outcomes, but they presented high uncertainty. Promoters might consider tentative recommendations of likely consequences that could be used to refine product development and clinical introduction. Recent preliminary CUAs considered the potential benefits of transfemoral and transtibial bone-anchored prostheses from the Australian government's prosthetic care perspective.^7,8,14,20–22^

### Emergence of preliminary CUAs

Concepts of preliminary CUAs are emerging.^1^ However, several obstacles must be overcome before preliminary CUAs are routinely performed by promoters of prosthetic care innovations. For instance, multiple pathways and disparity constructs make the outcomes of these CUAs (e.g., costs, utilities, ICURs) challenging to interpret (e.g., comparison between innovations, generalization across healthcare), appraise (e.g., Consolidated Health Economic Evaluation Reporting Standards (CHEERS), Consensus Health Economic Criteria (CHEC) extended checklists) and share (e.g., publication).^3,23–25^

Promoters rely on their abilities to make valid assumptions while opting for a specific CUA pathway of innovations.^1^ However, this does not mean that preliminary CUAs of a given innovation must be highly individualized. Arguably, the organization of the delivery and assessment of prosthetic care might be sufficiently transferable across innovations to consider a uniform approach to preliminary CUAs. ^7,8,14,20^

### Need for a basic framework of a preliminary CUA

The shortcomings of preliminary CUAs and the standardization of prosthetic care create opportunities for a basic framework of preliminary CUAs. Such a framework should be built based on fundamentals, applied principles of health economics, and recent preliminary CUAs of socket-free attachment for transfemoral and transtibial prostheses.^7,8,14^

The primary purpose of this article was to introduce a basic framework including a 15-step iterative process focusing on feasibility, constructs, analysis, and interpretations of outcomes of a preliminary CUA of prosthetic care innovations. Practically, a series of constructs and hands-on ways to gather information was presented. Furthermore, I recommended some facilitators transferable across preliminary CUA of prosthetic care innovations.

The secondary purpose was to appraise this basic framework considering potential strengths and weaknesses, barriers and facilitators, and returns on investment of the proposed preliminary CUA.

## BASIC FRAMEWORK

**Figure 1:**
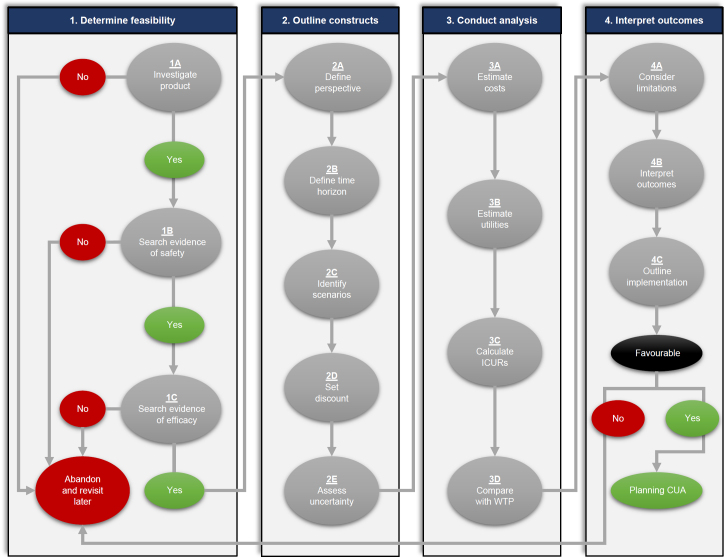
Overview of basic framework of preliminary cost-utility analysis (CUA) of prosthetic care innovations including on a 15-step iterative process. ICUR: Incremental cost-utility ratio, WTP: Willingness-to-pay threshold.

An overview of the iterative process of the basic framework of a preliminary CUA of a prosthetic care innovation is presented in Figure 1. This preliminary CUA was designed to compare the costs and utilities before or without (usual intervention) and after or with an innovation suitable to improve prosthetic fittings (new intervention).^4–6^

Next, all 15 steps of the process were individually presented, including a brief description of the concept, the specific aim, and some recommendations on ways to proceed that could facilitate the process when needed. Some barriers and facilitators were presented for the sake of completion, although they were basic and possibly evident for those astute in HEEs and CUAs (e.g., review literature, consult clinicians).^4–6^

Appraisal of the proposed preliminary CUA using the CHEERS and CHEC-extended checklists is detailed in the supplementary material.^23–25^

### Determine feasibility

This initial phase determines if the intended preliminary CUA is achievable depending on the strength of information available (Figure 1.1). This phase is organized around a three-step waterfall process with a decision point at every step to make sure resources are invested only if preliminary CUA is feasible. The analysis can stop at any step if the preliminary information is deemed unsatisfactory and could be revisited later on. Obtaining sufficient information leads to the next steps of the analysis.

### Investigate product (Step 1A)

This step gathers information about the clinical pathways of the innovation, including the technical description of the device and the surgical, medical, rehabilitation, and prosthetic care procedures like patient screening (e.g., clinical indications and contraindications), among others. Ultimately, this step reveals the unique value additions of the innovation compared to other interventions that could alleviate the current shortcomings of prosthetic fittings.^3,26–31^

Some obvious facilitators include the literature review and engagements with suppliers and clinicians to access guidelines and expert opinions.

### Search evidence of safety (Step 1B)

This step searches for what Ijzerman and Steuten (2011) called “likely safety” including some indications and preferably early evidence of the safety level of the innovation reported in terms of adverse events.^1^ Socket-based solutions should report the incidence of pain, slippage, pistoning, skin damages (e.g., allergies), and falls, to name a few.^29,32–36^ Socket-free innovations involving endo-skeletal osseointegrated implants with or without percutaneous parts should report pain, falls, stoma and soft tissue inflammation, loosening, periprosthetic fractures, breakage of implant parts, deep and superficial infections, intake of antibiotics, and removal of the implant.^37–39^ Levels of evidence (i.e., Level I-VII) and knowledge gaps should be considered when deciding the worthiness of the findings.

Preliminary CUA should be typically conducted shortly after commercialization when clinical use remains limited to a small group of patients. Therefore, evidence of safety for large cohorts (e.g., statistical power) over an extended observational study (e.g., several years follow-up) produced by independent parties might be desirable but unlikely. Alternatively, early evidence provided by manufacturers outside or within a registered clinical trial is expected. Level VI (e.g., single descriptive or qualitative study) or even Level VII (e.g., opinion of authorities and/or reports of expert committees) of evidence could be found in this step. Contemplating indications of the innovation's safety with a benevolent outlook is acceptable considering that only the safety prospect should be deemed sufficient to lead to the next step.

### Search evidence of efficacy (Step 1C)

This step searches for what Ijzerman and Steuten (2011) also called “likely efficacy,” including indications and, preferably, early evidence of the efficacy of the innovation.^1^ Efficacy includes, amongst others, self-reported satisfaction (e.g., Orthotics and Prosthetics Survey, Quebec User Evaluation of Satisfaction with Assistive Technology, socket prosthetic comfort score), the performance of physical tasks (e.g., Berg Balance Scale, timed get up and go, walking speed, two- or six-minute walk tests, functional ambulation profile, amputee mobility predictor with prosthesis), and specific (e.g., Questionnaire for Persons with a Transfemoral Amputation) and generic (e.g., 36-Item Short Form Survey (SF36), EuroQol-5 Dimension (EQ-5D)) health-related quality of life indicators (e.g., Quality-Adjusted Life Year, Disability-Adjusted Life Year).^33,34,36,40–42^

Evidence of efficacy might be easier to find because manufacturers tend to assess the benefits of innovations before the harms. Nonetheless, finding strong evidence of efficacy might be challenging for the same reasons indicated in Step 1B.

Levels of evidence (i.e., Level I-VII) and knowledge gaps should be considered when deciding the value of the finding. A critical facilitator is the health-related quality of life data review that can be readily mapped into QALY (e.g., SF36, EQ-5D).^43^ The absence of convertible health-related quality of life data is likely to stop the preliminary CUA because completion of Step 3B would be impractical. Any datasets that can be used to create either a baseline or incremental utility or both utilities with the innovation should be considered (e.g., an estimate of gain post-intervention).

### Outline constructs

This five-step phase defines the list of typical parameters framing a preliminary CUA (Figure 1.2).

### Define perspective (Step 2A)

Preliminary CUAs can be conducted from a broad taxpayer or healthcare perspective.^6,15^ However, surgical, medical, and prosthetic care costs are often undertaken in whole or in part by tertiary, primary, and secondary or allied health care services of government healthcare organizations or private companies operating together or separately.^2,15,20,44–46^ All costs are rarely collected in whole and reported to relevant services using a single integrated financial system. This step determines which perspective might be the most sensible, considering that preliminary CUAs can focus on a reasonably narrow perspective. Considering a government prosthetic care perspective to perform a preliminary CUA of a prosthetic care innovation seems indicated.^7,8,14,21^

### Define time horizon (Step 2B)

The length of time over which the innovation outcomes can be evaluated is called the time horizon. Choosing the appropriate time horizon can be problematic.^47,48^ This step aims at finding a compromise around a time horizon that is long enough to provide realistic and most probable intended benefit with the least approximation errors.^15,16,47–53^ Funding cycles of a preliminary CUA of an innovation advancing prosthetic fittings should consider the lifetime of the prosthetic components (e.g., socket, artificial joints).^7,8,14^

I suggest that a suitable compromise might be, at least, six years, because of the predictability of costs and the lifetime of components (e.g., two cycles of three years for a foot, three cycles of two years for a knee).^7,8^

### Identify scenarios (Step 2C)

Scenarios are commonly used to characterize the consequences of interventions for various health states and specific cases. Improvement in functional outcomes is often a consequence of choice. Level of function can be assess using Medicare Functional Classification Level (K-level). In principle, up to 15 scenarios can be considered when comparing possible progressions across the five K-levels ranging from K0 to K4 with and without the innovation (Table 1). This step identifies a limited series of scenarios that are the most plausible.

**Table 1:** Matrix of 15 possible scenarios comparing progressions of functional outcomes across the five Medicare Functional Classification Level (K-level) ranging from K0 to K4 without (usual) and with (new) intervention that could be considered in Step 2C.

New						
		K0	K1	K2	K3	K4
Usual	K0	scenario 1	scenario 2	scenario 3	scenario 4	scenario 5
	K1	-	scenario 6	scenario 7	scenario 8	scenario 9
	K2	-	-	scenario 10	scenario 11	scenario 12
	K3	-	-	-	scenario 13	scenario 14
	K4	-	-	-	-	scenario 15

I recommend exploring between three and five realistic scenarios including, the best, base, and worse cases depending on foreseeable costs and utility consequences of the innovation.^7,8^

### Set discount (Step 2D)

Discounting is the process of reducing future values of costs and utilities to their present values.^48^ The standard practice for full CUA is to discount values at 3% over the time horizon.^48^ This step ascertains the extent to which this rate should apply to the intended preliminary CUA.

I consider that no discount might be applied when the time horizon is reasonably short (e.g., up to six years) and the highest costs of the intervention are spent in the first few years of the funding cycle.^7,8^

### Assess uncertainty (Step 2E)

Estimates of costs and utilities are subjected to uncertainty depending on the sources of the data. Comprehensive CUA involves complex Markov-state transition models designed to investigate the impact of cost and utility estimates and provide parameters, models, and generalizable uncertainties.^15,16,50,51,53^ The sensibility of the outcomes of these models is also considered based on the probability of occurrence of events that might affect the analysis.^15^ This step aims to limit uncertainty by considering a limited number of practical events or health states.

I recommend making conservative assumptions that the innovation marginally improves the prosthetic fittings (e.g., reduce socket fittings by only one per annum). Considering multiple events or health states is beyond the scope of this analysis (e.g., reduce socket fittings by two, three, or more per annum).

Uncertainty of cost information of real and estimated costs, extracted from the schedule and financial records, can be reported using a variable called “prediction” presented in Frossard et al. (2018, 2020).^7,8^ This variable corresponds to the relative real over the total costs. A prediction of 0% and 100% indicated that the total cost is fully extracted from schedules and financial records, respectively.

Sensibility of datasets and the outcomes can be reported using basic descriptive statistics (e.g., mean, standard deviation, coefficient of variation, median, interquartile range, 95% confidence intervals, minimum, maximum, range).^7,8,14^ In some cases, inter-participant variability of costs might be reported using the coefficient of variation, where coefficients inferior to 33%, between 34% and 66%, and superior to 66% indicate low, moderate, and high inter-variability, respectively.^8^

### Conduct analysis

This four-step phase estimates costs, utilities, and ICURs based on the constructs determined in the previous phase (Figure 1.3).

### Estimate costs (Step 3A)

Ideally, actual labor and parts costs of prosthetic care with usual intervention and the innovation, can be extracted from financial systems for the largest possible cohort of participants. However, only partial information on the primary post-treatment costs over the time horizon might be available (Steps 1C, B).

I advise considering generic costs organized in schedules of allowable expenses.^14,20^ These schedules can be used, in part or whole, to estimate the most probable costs for prosthetic care without or with the innovation.^7,8,14^ A schedule is a matrix that presents costs at the intersection list of tasks in rows and the timeline of interventions between the columns.^3^ The type of tasks and frequency of interventions should be based on the standard of care recommended by clinicians and government agencies.^20,54,55^ The actual costs of labor and parts should be consistent with allowable expenses supported by reimbursement schemes (e.g., L-Codes), particularly when analyzing from the healthcare perspective. Examples of schedules of allowable expenses used for preliminary CUAs of lower limb bone-anchored prostheses can be used as a template.^7,8,14^

Healthcare organizations tend to provide support for categories of components depending on functional levels (e.g., K-Levels).56 Here, prosthetists are free to prescribe a model and brand according to the patients' specific needs. Thus, allocating lump sums rather than price tags for specific prosthetic components may be more acceptable. In all cases, I recommend presenting the source and analysis of datasets (e.g., actual vs. estimated). The series of assumptions made to estimate costs must be justified (e.g., hours of labor for socket fittings, frequency of replacement of prosthetic components).^14,20^

A conservative method to estimate costs is to assume that full allowable expenses are claimed, although some users might choose to keep components even after the warranty period or discard cosmetic covers. Alternatively, I consider that all prosthetic tasks are performed by qualified prosthetists even if some tasks might be undertaken by a technician working at a lower hourly rate. However, these suggestions could be discarded, particularly when the analyses focus on innovations improving service delivery.^57^

### Estimate utilities (Step 3B)

Primary utilities measured for groups of participants without and with the innovation are preferable, depending on the relevant healthcare organization. However, like costs, primary utilities collected at the outset of the preliminary CUA are likely to be unattainable.

Alternatively, I suggest using health-related quality of life data identified in Step 1C to estimate baseline and incremental utilities published together or separately. For instance, the dataset from SF36 can be converted into QALY applying the Ara and Brazier regression model used by Frossard et al. (2018).^7,8,43^ Plausible estimates of incremental utilities could be based on the assumption that users of the innovation are likely to experience a gradual gain of QALY between the worse, base, and best cases scenarios.^7,8^

I recommend describing the sources and processing of datasets, including the criteria to select publications and summarize the study designs used to measure original primary utilities. Gain of utilities should also be justified.

### Calculate incremental cost-utility ratios (Step 3C)

This preliminary CUA comes together when ICURs are calculated using the formula ICUR = (Costs with innovation – Costs with usual intervention)/(Utility with innovation – Utility with usual intervention).^4–6^ ICUR should be calculated for each scenario and plotted on a conventional four-quadrant cost-utility plane diagram indicating if the provision of the prosthetic care with innovation is more costly and more effective (Quadrant A: Consider ICUR), more costly and less effective (Quadrant B: Dominated), less costly and less effective (Quadrant C: Consider ICUR), and less costly and more effective (Quadrant D: Dominant) than usual intervention.^4–6^ I advise considering an indicative ICUR corresponding to the base-case scenario.^7,8^

### Compare with the willingness-to-pay threshold (Step 3D)

Typically, understanding outcomes of a CUA involved comparing ICUR and WTP. The oft-cited WTP is approximately $50,000 per QALY, depending on healthcare organizations.6 Based on figures frequently considered to determine the likelihood of adoption of an intervention, an indicative ICUR costing less than $20,000, between $20,000 and $100,000, and more than $100,000 per QALY could make the innovation most likely, likely, and unlikely, respectively, to motivate promoters to continue further product development and clinical introduction of the innovation.^6^ I advise a conservative WTP threshold of up to 20% lower than the recommended WTP (e.g., $40,000 per QALY).^7,8^

### Interpret outcomes

This four-step phase ascertains the extent to which the understanding of the outcomes of this preliminary CUA is sufficient to facilitate or curtail further product development and clinical introduction of the innovation (Figure 1.4).

### Consider limitations (Step 4A)

This step recognizes the impacts of assumptions on the overall outcomes of the analysis. The typical and specific limitations of calculations of ICURs are discussed (e.g., mismatching datasets).

I suggest exploring possible causes of cost overestimation (e.g., claiming full allowable expenses, tasks only performed by qualified prosthetist) and utilities underestimations (e.g., low incremental gains, consider utilities gained post-treatment consistent over time). I recommend acknowledging the extent to which the aggregate ICURs mismatched data (e.g., sources, jurisdictions, onset, post-operative timeline).^7,8^

### Interpret outcomes (Step 4B)

This step considers the cost-effective conditions for the innovation. It ascertains by how much the QALY must be increased to offset its costs, and the requirements to make the indicative ICUR below WTP.

I advise interpreting the analysis outcomes after comparing the costs, utilities, and ICURs with other competing interventions that could improve prosthetic fittings. Potential generalization of the outcomes should be investigated, considering the limitations. Finally, I advise basing the recommendations for wider clinical usage and likelihood of adoption of the innovation on the figures presented in Step 3D.

### Outline implementation strategy (Step 4C)

This last step examines the innovation against other interventions and relevant healthcare cost-utility standards. Decision-makers should comprehensively gauge whether the outcomes produced were robust enough to justify pursuing subsequent implementation strategy. Weak or unfavorable outcomes might encourage innovators to rethink product development and revisit opportunities for preliminary CUAs at a later stage. Strong and favorable outcomes might warrant continuing further product development and clinical promotion of the innovation.

I suggest highlighting the elements of the preliminary CUA that could facilitate the design of the potential primary or modelling CUAs of the innovation (e.g., within-trial and beyond-trial horizon studies).^47^ Regardless of the recommendations, I advise outlining subsequent implementation strategies that could be deemed outside the scope of the analysis and the purpose of another process.

## APPRAISAL OF BASIC FRAMEWORK

### Strengths and weaknesses

The proposed basic framework will provide timely information. This preliminary CUA will generate sufficient evidence to identify gaps in evidence and improvement, educate about the design of primary or modeling studies, and fast-track approval of innovation from governing bodies.

This framework carries the intrinsic limitations of the usual preliminary CUAs mentioned in Frossard (2021).^3^ The inherent limitations to the analysis include narrow perspective, plausible scenarios, and mid-term time horizon. The cost and utility estimates are built around best-known evidence and substantial assumptions. ICURs would be based on mismatched costs and utilities. Reporting of the uncertainty and sensibility data will likely lack definition.

Altogether, I predicted that the analysis might have a weak, moderate, and strong capacity to address 9 (33%), 8 (30%), and 10 (37%) of the items in the CHEERS checklist, including 7 (44%), 6 (38%) and 3 (19%) of the items in the Methods, and 2 (40%), 2 (40%), and 1 (20%) of the items in the Results sections, respectively.^23,24^ The proposed preliminary CUA might be capable and incapable of addressing items 11 (58%) and 8 (42%) in the CHEC-extended checklists, respectively.^24,25^

### Barriers and facilitators

As outlined in Table 2, I identified a total of 25 barriers that could be overcome by 52 facilitators likely to be transferable across preliminary CUAs of prosthetic care innovations. I believe these key but not comprehensive recommendations can be included:

- Choosing abided constructs. The preliminary CUA design (Step 2), particularly the time horizon, emanated from educated choices integrating various basic and applied CUA methodological approaches presented in guidelines and recent publications.^48^ I recommended considering constructs that are consistent with recent preliminary CUA socket-free solutions.^7,8,14^ Choosing similar constructs would significantly streamline decision-making in all Steps 2A, B, C, and D. This should greatly facilitate the interpretation of the outcomes (Step 4B) and the gauging of the value proposition of the innovation compared to other interventions (Step 4C). This difference in outcomes between analyses could be minimally attributed to confounding constructs.
- Building on prior schedules of expenses. The cost estimates (Step 3A) could be largely guided by an initial template of schedules considering the prosthetic care provision costs for lower limb socket-suspended and bone-anchored prostheses.^7,8,14^ Some generic tasks and timeline of interventions could be transferable. Other costs specific to each innovation must be tabulated into the new schedules recommended by clinicians and government agencies.
- Capitalizing on benchmark baseline and incremental utilities. The utility estimates (Step 3B) could be educated by benchmark baseline and incremental utilities provided in the health economic literature focusing on socket-based or socket-fee prostheses.^10-13,15-19,57^

**Table 2:** Overview of manageable barriers and transferrable facilitators of basic framework of preliminary cost-utility analysis (CUA) comparing usual intervention (e.g., socket-based, socket-free using osseointegration) and a prosthetic care innovation susceptible to improve prosthetic fittings. ICUR: Incremental cost-utility ratio, K-level: Medicare Functional Classification Level, QALY: Quality-adjusted life-year, $: Australian Dollar.

Barriers	Facilitators
**1. Determine feasibility**	
Investigate product (Step 1A)	
1. Understand the technical description of the treatment (e.g., identify parts)	1. Find technical information provided by the supplier of the innovation (e.g., website, flyer)
2. Understand the surgical, medical, rehabilitation and prosthetic care procedures (e.g., Understand clinical indications and contraindications)	2. Find clinical guidelines for the prescription of the innovation provided by the supplier 3. Seek opinion of expert clinicians about indications and contraindications
3. Understand unique added value of the innovation compared to other interventions	4. Extract information provided in publications about innovation 5. Identify uniqueness of the treatment after cross-comparison with other interventions 6. Establish if the potential benefits of the innovation justified investigating safety
Search evidence of safety (Step 1B)	
4. Foresee indications of safety of the innovation	7. Sass out potential harms of innovation 8. Compare potential risks with other interventions
5. Find early evidence of safety of the innovation	9. Search literature focusing on safety of the innovation 10. Review level of evidence of adverse events (i.e., Level I-VII, registered clinical trial)
6. Identify evidence gaps about safety of the innovation	11. Acknowledge evidence gaps about safety of the innovation 12. Establish if evidence of safety of the innovation justified investigating efficacy
Search evidence of efficacy (Step 1C)	
7. Foresee indications of efficacy of the innovation	13. Sass out potential benefits of innovation 14. Compare potential benefits with other interventions
8. Find evidence of the efficacy of the innovation	15. Search literature focusing on efficacy of the innovation 16. Review level of evidence of satisfaction, function (e.g., performance of physical tasks) and health-related quality of life (i.e., Level I-VII, registered clinical trial)
9. Identify evidence gaps about efficacy of the innovation	17. Acknowledge evidence gaps about efficacy of the innovation 18. Establish if health-related quality of life data of the innovation is sufficient to justified continuing CUA
**2. Outline constructs**	
Define perspective (Step 2A)	
10. Choose healthcare perspective considering surgical, medical and prosthetic care expenses	19. Accept that considering whole care expenses together might have little relevance, in fine, because of the separation between primary, secondary, and tertiary services in typical healthcare systems 20. Simplify analysis be considering only a prosthetic care perspective
Define time horizon (Step 2B)	
11. Find the time horizon that is long enough to provide realistic outcomes but the least subjected to large approximation errors	21. Understand that prediction of costs of prosthetic components over the long period of time is more likely to be grossly inaccurate 22. Acknowledge studies suggesting that a rather short time horizon would be indicated for the preliminary analysis 23. Consider that six-year time horizon might be a suitable compromise because of the predictability of costs and lifetime of components
Identify scenarios (Step 2C)	
12. Identify a small series of plausible scenarios	24. Consider 15 scenarios for all possible progressions across K-levels 25. Select up to five realistic scenarios most likely to represent expected clinical outcomes with the innovation including worse, best and base cases
Set discount (Step 2D)	
13. Ascertain to which extent typical discounting rate should apply	26. Consider applying no discount when time horizon is short enough to predict costs 27. Consider applying no discount when most important costs might occur at the beginning of the cycle
Assess uncertainty (Step 2E)	
14. Find ways to determine the uncertainty	28. Make the conservative assumption that the innovation would minimally improve prosthetic fittings 29. Consider that looking at multiple events or health states is beyond the scope of this analysis
15. Find ways to present the sensibility	30. Choose to report sensibility analysis using only basic descriptive statistics including coefficient of variation
**3. Conduct analysis**	
Estimate costs (Step 3A)	
16. Estimate costs for the provision of prosthetic care without and with the innovation	31. Acknowledge that primary costs with the innovation might not be available in relevant healthcare system 32. Create schedules of allowable expenses for labour and parts for the provision of prosthetic care without and with innovation 33. Apply costings recommended by the healthcare system
Estimate utilities (Step 3B)	
17. Estimates utilities experienced by users without and with the innovation	34. Acknowledge that primary utility data with the innovation might not be available for groups of users involved in relevant healthcare system 35. Search utility information with the innovation in the literature 36. Consider utility information published and convert data to create baseline utility reported in QALY 37. Assume that users experience a small increase of utilities with the innovation compare to baseline 38. Assume that utilities experienced without and with the innovation remain steady during the time horizon
Calculate ICURs (Step 3C)	
18.Determine which scenario could provide a tentative ICUR	39. Assume that the base-case scenario should be correspond to the indicative ICUR
Compare with WTP (Step 3D)	
19.Identify the sensible WTP commonly accepted in the relevant health care	40. Consider that a conservative WTP is $40,000 per QALY that is significantly lower that oft-cited WTP
20.Identify thresholds most likely to motivate promoters to continue the developments of the innovation	41. Consider that an indicative ICUR costing less than $20,000 per QALY is most likely to motivate promoters to continue the developments of the innovation
**4. Interpret outcomes**	
Consider limitations (Step 4A)	
21. Understand the effects of the series of assumptions	42. Concede that analysis is noticeably limited by a series of assumptions 43. Look at how costs might have been over-estimated 44. Look at how utilities might have been under-estimated 45. Acknowledge when ICURs aggregates mismatched data
Interpret outcomes (Step 4B)	
22. Assess how the treatment compared to other interventions	46. Estimate the range of costs and utilities that might be required to make the innovation cost-effective and below WTP 47. Determine if the innovation has the potential to be more cost-effectiveness than competing interventions
23. Assess limitations to generalization of the outcomes	48. Concede that generalization of outcomes might be limited
Outline implementation strategy (Step 4C)	
24. Gauge the worthiness of data to justify introducing of the innovation in healthcare	49. Establish how the indicative ICUR with the innovation stacks up against other interventions 50. Decide if the analysis provided sufficient evidence to motivate promoters to encourage clinical adoption in relevant healthcare
25. Identify how information gathered during this analysis could inform the design subsequent full CUA of the innovation	51. Acknowledge that outline pathways for the clinical introduction of the innovation is beyond the; scope of this analysis 52. Detail how this information provided can inform subsequent primary and modelling CUAs of the innovation

### Returns on investment

Questions might be raised about the returns on investment of the proposed preliminary CUA. Although some safeguards were embedded into the initial feasibility phase to curtail unnecessary work, the entire preliminary CUA requires noticeable efforts depending on the source of data (e.g., design schedules, extract costs, map utility).

The returns might be unclear because of the important structural uncertainty, medium grade of evidence, and tentative recommendations.^1^

However, policymakers in the healthcare sector might see some benefits of systematically embedding such preliminary CUA into their horizon scanning process.1 It can contribute to deciding whether a new prosthetic care intervention shows early signs of cost-utility. Promoters of new interventions might deem this preliminary CUA a worthwhile investment to support applications for healthcare approval.^2^

## CONCLUSIONS

This study was an initial effort to standardize a basic framework of preliminary CUA comparing the prosthetic care provisions with and without innovation suitable to improve prosthetic fittings. This new approach to preliminary CUA has the potential to simplify the selection of methods, standardize outcomes, ease comparisons between innovations and streamline pathways for adoption while facilitating the production of a body of literature on prosthetic health economics. Insights into the next phase of development of this method might come from Masaaki Imai, a Japanese organizational theorist, and management consultant. He stated that it is impossible to improve any process until it is standardized. He added that if the process is shifting from here to there, then any improvement will just be one more variation that is occasionally used and mostly ignored. One must standardize, and thus stabilize the process, before continuous improvement can be made. Therefore, I welcome further experiments of this proposed analysis with emerging prosthetic care innovations. This will refine and validate the standard basic framework of preliminary CUA. Hopefully, this collegial effort will facilitate the adoption of economic prosthetic care innovations that could improve the lives of individuals suffering from limb loss worldwide.

## CALL TO ACTION

- Continue the discussion between promoters of prosthetic care innovations around the use and validation of preliminary CUAs framework.
- Inspire authors of health economic evaluations to road-test the proposed framework with a series of emerging prosthetic care innovations susceptible to improve prosthetic fittings.
- Encourage authors of health economic evaluations of prosthetic care innovations to capitalize on the benefits of early and preliminary CUAs during development of the innovations.

## DECLARATION OF CONFLICTING INTERESTS

The author is in the view that these competing interests do not conflict with the content of this manuscript. Laurent Frossard, Director and Chief Scientist Officer of YourResearchProject Pty Ltd, has worked as consultant for several organisations on non-related educational programs and projects of research focusing on recording loading data, developing of database to record clinical outcomes as well as drafting grants and manuscripts for Cognitive Institute, Exercise & Sports Science Australia, Griffith University, iPug Pty Ltd, Middlesex University, New Zealand Artificial Limb Service, Osseointegration Group of Australia Pty Ltd, OSSUR, Poly-Orthodox International, Queensland Artificial Limb Service, Queensland University of Technology, Return to Work-South Australia, South Australia Health, Tequir S.L, University of the New South Whales, University of the Sunshine Coast.

## SOURCES OF SUPPORT

This study was funded by YourResearchProject Pty Ltd.

## LIST OF ABBREVIATIONS

CHEC: Consensus Health Economic Criteria extended checklist
CHEER: Consolidated Health Economic Evaluation Reporting Standards checklist
CUA: Cost-utility analysis
EQ-5D: EuroQol-5 Dimension
ICER: Incremental cost-effectiveness ratio
ICUR: Incremental cost-utility ratio
K: Medicare Functional Classification Level
QALY: Quality-adjusted life-year
SF36: 36-Item Short Form Survey
WTP: Willingness-to-pay threshold
